# Worldwide increased prevalence of human adenovirus type 3 (HAdV-3) respiratory infections is well correlated with heterogeneous hypervariable regions (HVRs) of hexon

**DOI:** 10.1371/journal.pone.0194516

**Published:** 2018-03-28

**Authors:** Ezazul Haque, Urmila Banik, Tahmina Monwar, Leela Anthony, Arun Kumar Adhikary

**Affiliations:** 1 Unit of Microbiology, AIMST University, Faculty of Medicine, Jalan Bedong Semeling, Bedong, Kedah, Malaysia; 2 Unit of Pathology, AIMST University, Faculty of Medicine, Jalan Bedong Semeling, Bedong, Kedah, Malaysia; 3 Unit of Community Medicine, AIMST University, Faculty of Medicine, Jalan Bedong Semeling, Bedong, Kedah, Malaysia; Universidad Nacional de la Plata, ARGENTINA

## Abstract

Human adenovirus type 3 (HAdV-3) respiratory infections occurs worldwide in both children and adults, leading to severe morbidity and mortality, particularly in the paediatric age group and especially in neonates. During HAdV infection, neutralizing antibodies are formed against the epitopes located in the hyper variable regions (HVRs) of the hexon protein. These neutralizing antibodies provide protection against reinfection by viruses of the same type. Therefore it is reasonable to speculate that variations of HAdV-3 in the HVRs could impair the immunity acquired by previous infection with a different strain with variation in its HVRs. HAdV-3 has recently become the major agent of acute respiratory infection worldwide, being responsible for 15% to 87% of all adenoviral respiratory infections. However, despite the increased prevalence of HAdV-3 as respiratory pathogen, the diversity of hexon proteins in circulating strains remains unexplored. This study was designed to explore the variation in HVRs of hexon among globally distributed strains of HAdV-3 as well as to discover possible relationship among them, thus possibly shedding light on the cause for the increased prevalence of HAdV-3. In this study, for the first time we analysed the hexon proteins of all 248 available strains of HAdV-3 from the NCBI database and compared them with those of the HAdV-3 prototype (GB stain). We found that the HVRs of HAdV-3 strains circulating worldwide were highly heterogeneous and have been mutating continuously since -their original isolation. Based on their immense heterogeneity, the strains can be categorized into 25 hexon variants (3Hv-1 to 3Hv-25), 4 of which (3Hv-1 to 3Hv-4) comprises 80% of the strains. This heterogeneity may explain why HAdV-3 has become the most prevalent HAdVs type worldwide. The heterogeneity of hexon proteins also shows that the development of a vaccine against HAdV-3 might be challenging. The data on hexon variants provided here may be useful for the future epidemiological study of HAdV-3 infection.

## Introduction

The 57 recognized types of human adenoviruses (HAdVs) have been classified into 7 species, A to G, on the basis of their serological, biochemical and genetic criteria; and 27 more types are waiting for formal recognition (http://hadvwg.gmu.edu/) [1]. HAdV-3 of species B is among the most common types implicated in endemic and epidemic HAdV infections in children and adults globally[2–6]. Occasionally this virus is associated with respiratory infections in the military trainees[4]. This clinically significant type is related to a broad spectrum of diseases including respiratory illnesses, epidemic keratoconjunctivitis, pharyngoconjunctival fever, and acute gastroenteritis [7–9]. HAdV-3 has a predilection for the pediatric age group; young children and neonates are particular targets of nosocomial as well as community-acquired pneumonia with high levels of morbidity and even mortality [2,10–14]. The sequelae of pulmonary infection due to HAdV-3 strains include bronchiectasis, bronchiolitis obliterans, unilateral hyperlucent lung, and persistent abnormal pulmonary function [15]. A survey from the from the World Health Organization (WHO) reveals that HAdV-3 was responsible for 13% of all adenoviral infections from 1967 to 1976 [3]. Over the last few years of HAdV-3 has become the major agent of acute respiratory infection worldwide and encompassing 15 to 87% of adenoviral respiratory infections [2,4–6,12,13,16–26].

HAdVs are non-enveloped viruses with linear dsDNA within an icosahedral nucleocapsid approximately 70–90 nm in diameter. The HAdV capsid is formed by three viral capsid proteins, namely the penton base, hexon, and fiber [27,28]. The fiber and penton are primarily responsible for the attachment and internalization, respectively, of the virus in the host cell. The hexon is the key structural protein in the icosahedral adenovirus capsid. The hexon of HAdV-3 (GB stains) is a 945 amino acid (AA) polypeptide encoded by 2835 nucleotides and extends from 18,420 to 21,254 base pairs (bps) in the HAdV-3 genome. [29,30]. The hexon capsomeres are packed tightly to form a protein shell that protects the inner components of the virion. The outward portion of the hexon protein is arranged in 3 loops (L1, L2, and L4).

Alignment of hexon protein sequences from different types of HAdVs has revealed that the amino acid (AA) sequences in the loops are highly variable in multiple locations; these have been designated as 7 hypervariable regions (HVR1 to HVR7). HVR1 to HVR6 are located in Loop 1; and HVR7 is located in Loop 2. Although AA sequences in HVRs are dissimilar in different HAdV types, they are usually similar in the different strains of an HAdV type. Type-specific epitopes are located in 1 or more HVRs of the hexon protein. During HAdV infection, antibodies are formed against the structural and non-structural proteins of the virion. But antibodies formed against the epitopes on hexon proteins provide protection against reinfection by a virus of the same type [29–33]. Therefore it is reasonable to speculate that the variation in HVRs of HAdV-3 could impair the pre-existing immunity acquired through previous infection by a different strain with variation in its HVRs. Consequently the HVRs of the hexon protein are the most important structural proteins of HAdV-3 for molecular analysis in order to elucidate the virological basis of the worldwide increased prevalence of HAdV-3 respiratory infection.

With this background in mind, the present study was designed to explore the variation of HVRs of hexons among globally distributed strains of HAdV-3 as well as to find possible relationship between the variability of HVRs of the hexon protein and the worldwide increased prevalence of HAdV-3 infection.

## Materials and methods

### Sample

HAdV-3 (GB strain) was purchased from the American Type Culture Collection (ATCC, Manassas, VA). In 1953, a prototype GB strain of HAdV-3 was isolated in the state of Maryland from the nasal washing of a male patient who was suffering from the common cold [34]. In our study HVRs encoding regions of hexon proteins of the GB strain were sequenced. The obtained sequence was used for BLAST (basic local alignment searching tool) search. The predicted hexon protein sequence of GB strain was compared with the other hexon protein sequences of HAdV-3 obtained from the NCBI database.

### Sequencing of hexon gene of GB strains of HAdV-3

#### Extraction of viral DNA

HAdV-3 DNA was extracted from the culture fluid following the manufacturer’s protocol (Norgen Biotek Corp., Thorold, ON, Canada); 150 μL of culture fluid was placed in a 1.5-mL microcentrifuge tube. “Digestion Buffer A” was added to adjust the volume to 300 μL. Then 12 μL of “Proteinase K” was added to the suspension, which was mixed by gentle vortexing and incubated at 55°C for 1 hour. After incubation, 300 μL of “Buffer SK” was added to the lysate, mixed, and vortexed. Then 300 μL of 90% “Ethanol” was added and mixed by vortexing. The spin column was then attached to a collection tube. The mixture was then placed in the spin column assembly. The column was capped and the unit was centrifuged for 3 minutes at 8000 rpm. After centrifugation the flowthrough was discarded and the spin column was reattached to the collection tube. The centrifugation step was repeated for the same period and at the same speed until all of the mixture had passed through the column. Then 500 μL “Wash Solution A” was placed in a column and centrifuged for 1 minute at 14,000 rpm. After centrifugation the flowthrough was discarded and the spin column was reattached to the collection tube. Then, again, 500 μL of “Wash Solution A” was placed in the column and centrifuged for 2 minutes at 14,000 rpm. The spin column was then carefully detached from the collection tube and the collection tube with flowthrough was discarded. The spin column (containing DNA bound to the resin) was assembled with a 1.7 mL “Elution tube.” Then 200 μL of “Elution buffer B” was added to the center of the resin bed and centrifuged for 1 minute at 6000 rpm. A portion of Elution buffer B passed through the column, allowing hydration of the DNA. It was then centrifuged at 14,000 rpm for 2 minutes and the total elution volume was collected. The quantity of the DNA was determined by spectrophotometry and stored at -20° C.

#### DNA amplification and sequencing

The primer for the HAdV-3 hexon protein comprises about 2.8 kbp. To amplify and sequence the hexon HVRs, the primers described by Takeuchi et al. were used [35]. The first set of primers included HX5-1 (forward primer), 5'-AAGATGGCCACCCCCTCGATGATGCCGCAGT-3', and HX3-1 (reverse primer), 5'-CACTTATGTGGTGGCGTTGCCGGCCGAGAACGG-3', which were designed to amplify the region that correspond to 1 to 2829 bp in the hexon base sequence of Ad type 3. Polymerase chain reaction (PCR) amplification was carried out in a 50-μL reaction mixture containing aliquots of 1 μL genomic DNA, 3 μL of each primer, 25 μL of PCR master mix, and 18 μL of nuclease-free water. Thermal cycling consisted of denaturation at 94°C for 1 minute, annealing at 40°C for 1 minute, and extension at 72°C for 2 minutes. In all 40 cycles were used in the PCR process. For negative control, 1μL distilled water was used.

**Fig 1:**
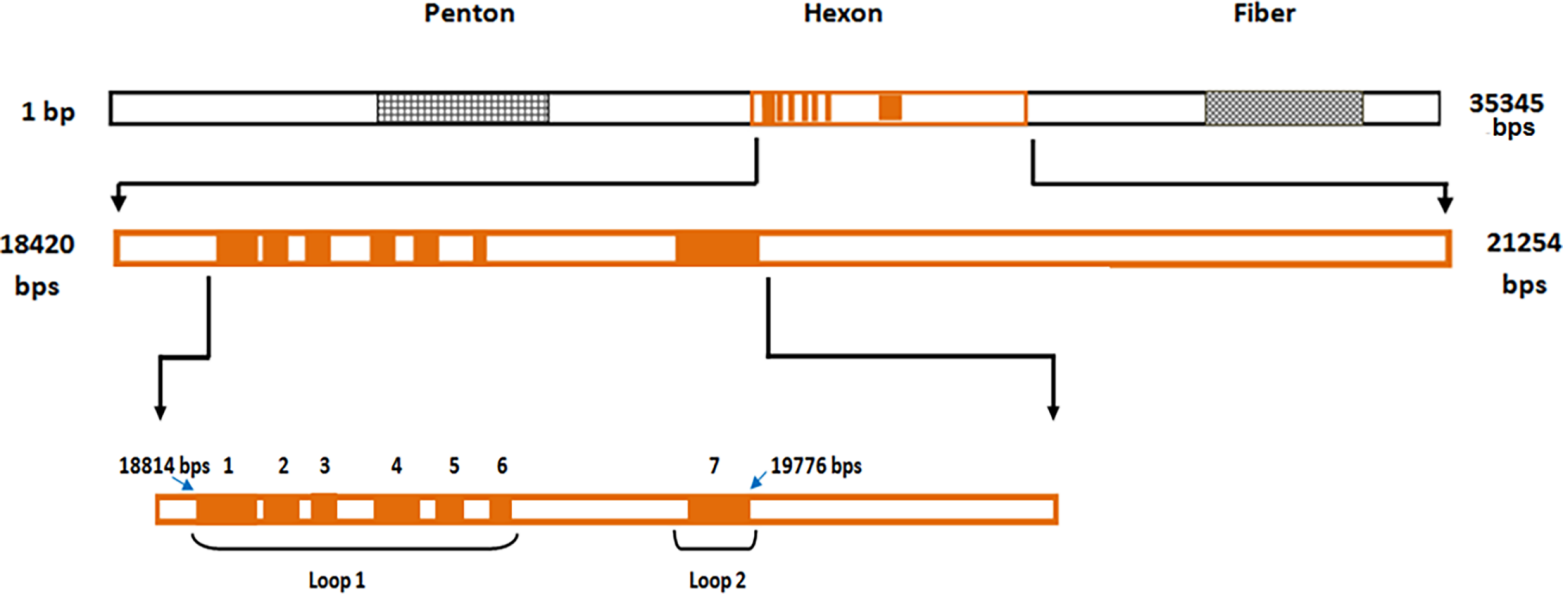
HAdV-3 genome and hexon gene hypervariable regions. The HAdV-3 GB strain genome is 35345 bp long (*Gen*Bank ID: AY599834). The hexon protein is a 945–amino acid (AA) polypeptide encoded by 2835 nucleotides that extends from 18,420 to 21,254 bp in the HAdV-3 genome. HVR1 to HVR6 are located in Loop 1 and HVR7 in Loop 2 of the hexon between 18,814 to 19,777 bp of the genome.

The sequence of HAdV-3 was elicited by using the primer HX 5–3 (forward primer: 5ʹ CAC ATC GCC GGA CAG GAT GCT TCG GAG TA 3ʹ) and HX 3–4 (reverse primer: 5ʹ GTG TTG TGA GCC ATG GGG AAG AAG GTG GC 3ʹ) (Fig 1). Sequence analysis was done with Genetyx (Genetyx Corporation, Tokyo) software; a 993 bp sequence containing the 7 HVRs was submitted to “DNA Data Bank of Japan” (DDBJ)/*Gen*Bank.

### Selection of HAdV-3 hexon gene sequences from the GenBank database

Selection of the HAdV-3 strains was done via the database of the National Center for Biotechnology Information (NCBI) (http://www.ncbi.nlm.nih.gov/). The nucleotide search option of *Gen*Bank and BLAST search were implemented. For BLAST search, a 993 bp nucleotide of a hexon gene of the GB strain that encodes the 7 HVRs of HAdV-3 was used. A manual search was done using *Gen*Bank’s nucleotide search option using the key words “adenovirus type 3 hexon gene.” All nucleotide sequences were saved in “FASTA” fromat as a text document. Then the nucleotide sequences of all the strains of the HAdV-3 hexon protein were translated into predicted amino acid (AA) by Genetyx (Genetyx Corporation, Tokyo) software. From the translated AA sequences only the strains that containing all 7 HVRs were selected for further analysis. Then all 248 AA sequences of hexon including the GB strain were copied and pasted together in FASTA format as a text document. The selected strains along with their *Gen*Bank accession numbers were tabulated according to their country of origin (S1 Table).

### Multiple sequence alignment of the HAdV-3 hexon

The AA sequences (containing all 7 HVRs) of the selected strains were aligned first by MEGA 7 using Clustal X to determine their identity or similarity (http://www.megasoftware.net). Then Gentyx was used to edit the 25 variants (39 strains).

#### Building alignment by MEGA

*Alignment Explorer* is launched by selecting the Align | Edit/Build Alignment on the launch bar of the main MEGA window. Then Create New Alignment was selected. From the dialogue box “Protein sequence alignment” was selected. From the Edit menu of Alignment Explorer insert sequence from the file option “HAdV-3 all hex” was selected. Then from the main menu of Alignment | Align by ClustalW was selected using the ClustalW algorithm. “Ok” button was clicked to accept the default settings for ClustalW. Once the alignment was complete, the alignment session was saved as HAdV-3 hex align. mss by selecting Data *|* Save Session from the main menu. From the alignment based on similarity and variation in HVRs with the GB hexon sequence the strains were categorized as variants and those variant were designated as as HAdV-3 hexon variant (3Hv).

#### Building and editing the alignment of selected variants by Genetyx

**Fig 2:**
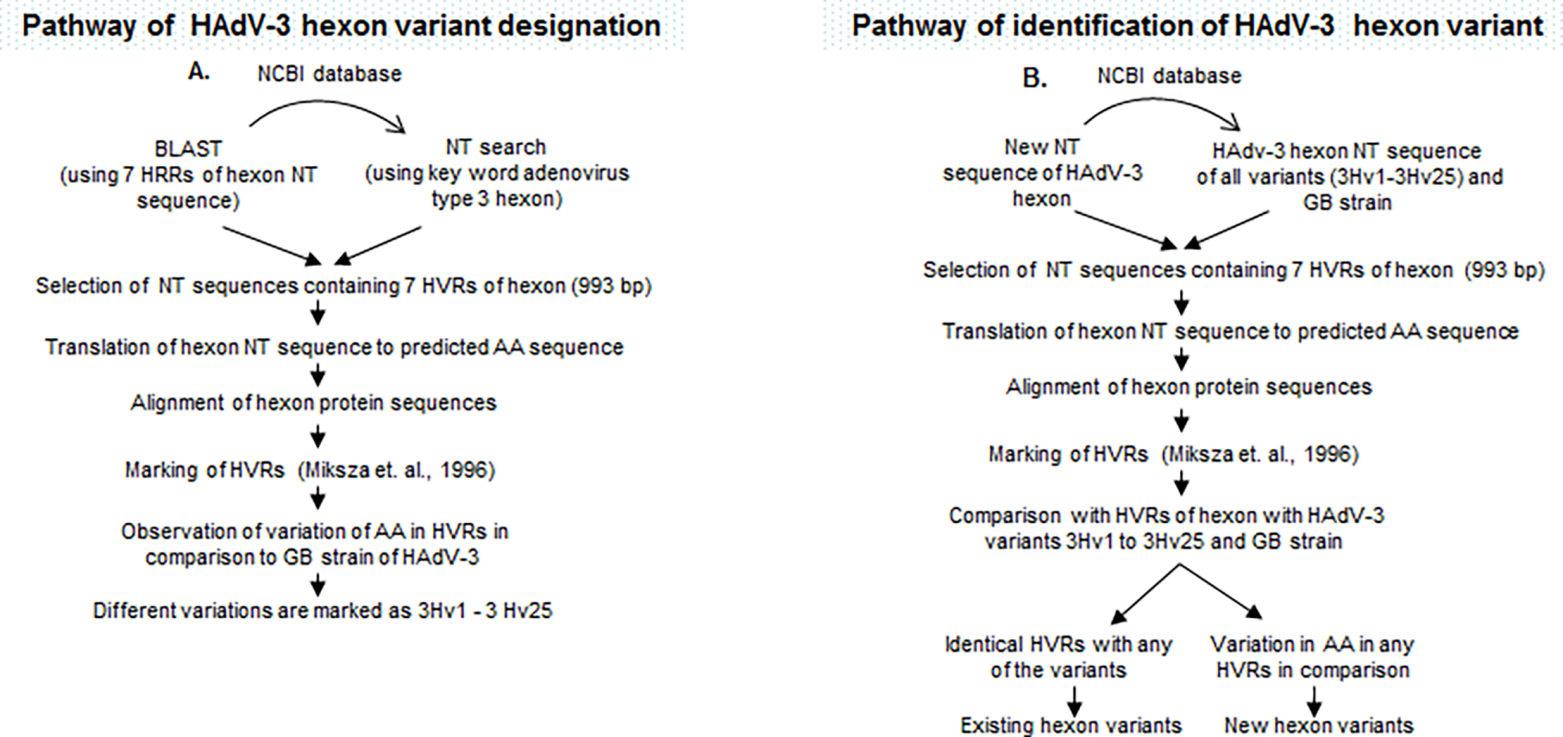
(A) Pathway of designation of human adenovirus type 3 hexon variants. (B) Method of identification of new hexon variants of human adenovirus type 3. HAdV-3-human adenovirus type 3, NT-nucleotide, BLAST-basic local alignment searching tool, AA-amino acid, HVRs- hypervariable regions, 3Hv-hexon variant of adenovirus type 3.

From all the hexon variants 1 reprentative was selected; futher alignment was made by Genetyx for the convenience of editing and publishing. In short, from the file option of the menu bar multisequence was selected. Then, from the multifiles selector, amino acid sequence was selected and alignment was done. From that alignment the intervening regions between the HVRs were deleted for better reproduction. The procedure for designating HAdV-3 hexon variants and their future identification are illustrated by the flowchart in Fig 2.

## Results

### GenBank submission

The nucleotide sequence of the GB strain encoding the HVRs of HAdV-3 hexon protein was 993 bp in length. The sequence was deposited in *Gen*Bank/DDBJ under the accession number LC159539. The amino acid sequences of the residues were deduced (S1 Fig).

### Blast search

From BLAST search, 98 items (strains) were identified. However, a manual search using the nucleotide search option from *Gen*Bank showed more than 800 items. It appears that the lower number of items obtained by BLAST is due to highly heterogeneous HVRs of HAdV-3. From both the BLAST search and nucleotide search of GenBank a total of 248 strains were selected that contained all 7 HVR sequences of the hexon. Analysis revealed that 248 strains were dispersed in 7 countries worldwide. The highest number of nucleotide sequence (81) were acquired from China, followed by Japan (52), Taiwan (49), Germany (36), Korea (25), the United States (3) and India (2)

**Fig 3:**
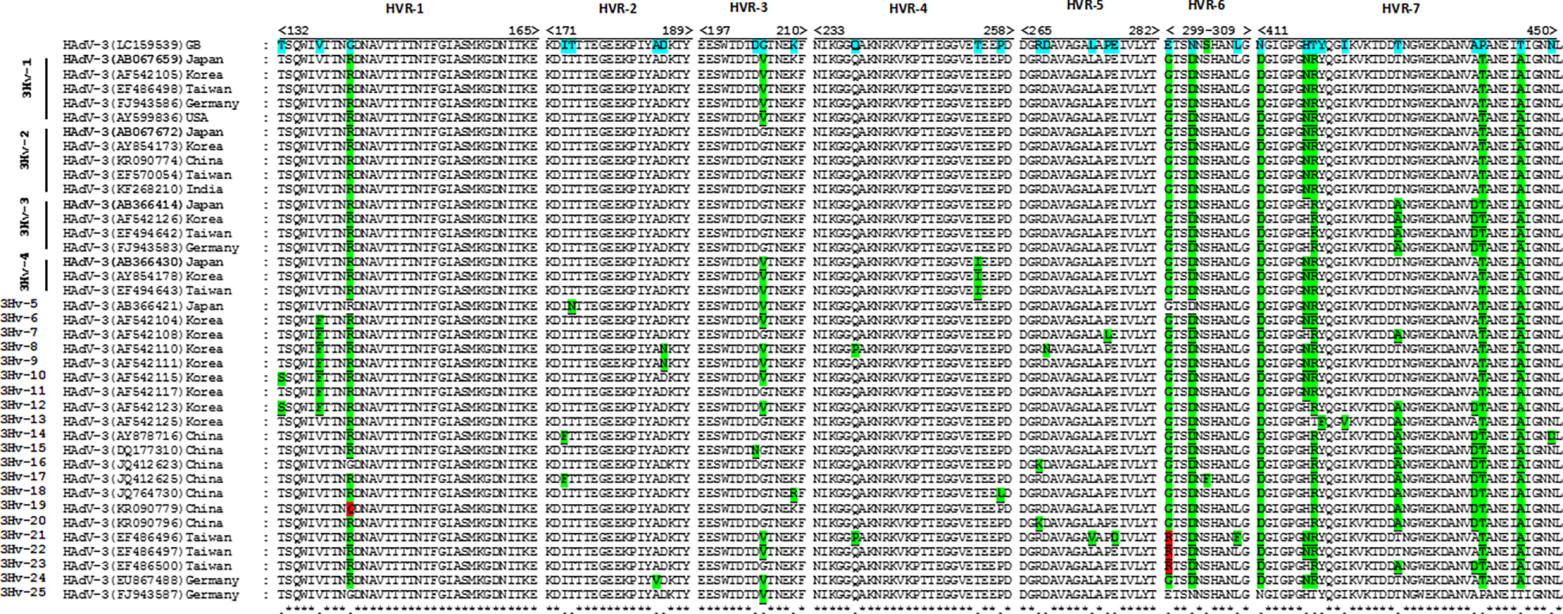
Comparison of the predicted amino acid sequences of 7 HVRs of 25 hexon variants (3Hv-1 to 3Hv-25) of HAdV-3. The variant strains were compared with the GB strain. Because 3Hv-1 to 3Hv-4 together comprise the majority (80%) of the variants, one representative strain from each country was selected. One reprentative strain from 3Hv-5 to 3Hv-25 was selected. The AA sequences were aligned by Genetyx software (Genetyx Corporation, Tokyo). The hypervariable regions were marked manually following the description by Miksza et al. (1996). The intervening AA sequences between the HVRs were deleted after alignment. The homology of AAs with GB strains is indicated by an asterisk (*) as well as by a green or red shadow, whereas the variations are indicated by an underscore (_).

**Fig 4:**
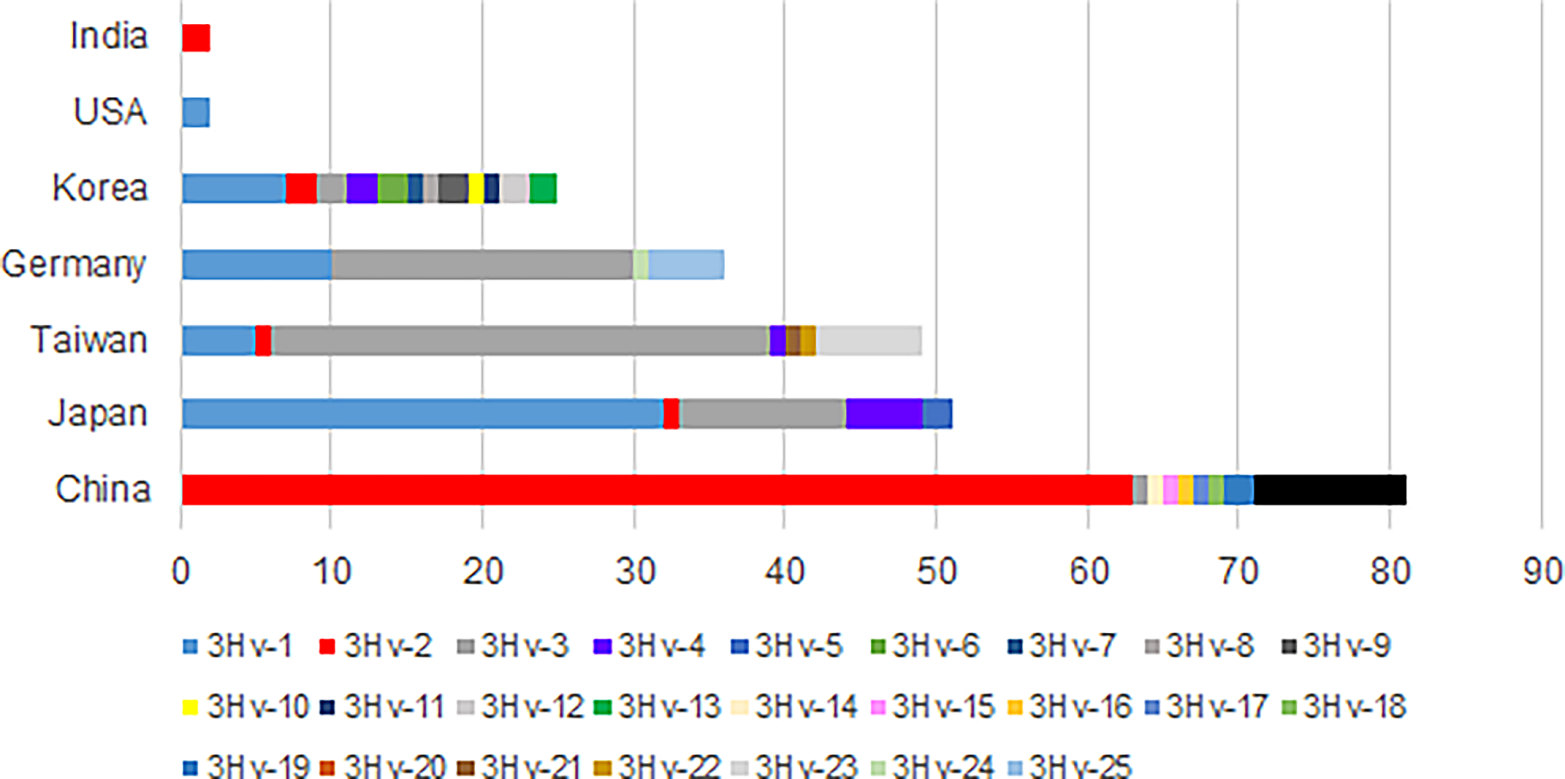
Country-based distribution of HAdV-3 hexon variants. The country is indicated by a bar and the variants are indicated by different colors shown below the lowest bar. Korea has the highest number of variants (12 variants within 25 strains). This is followed by China (9 variants within 81 strains), Taiwan (7 variants with 49 stains), Japan (5 variants within 52 strains), Germany (4 variants within 36 strains). The United States and India each have only 1 hexon variant.

**Fig 5:**
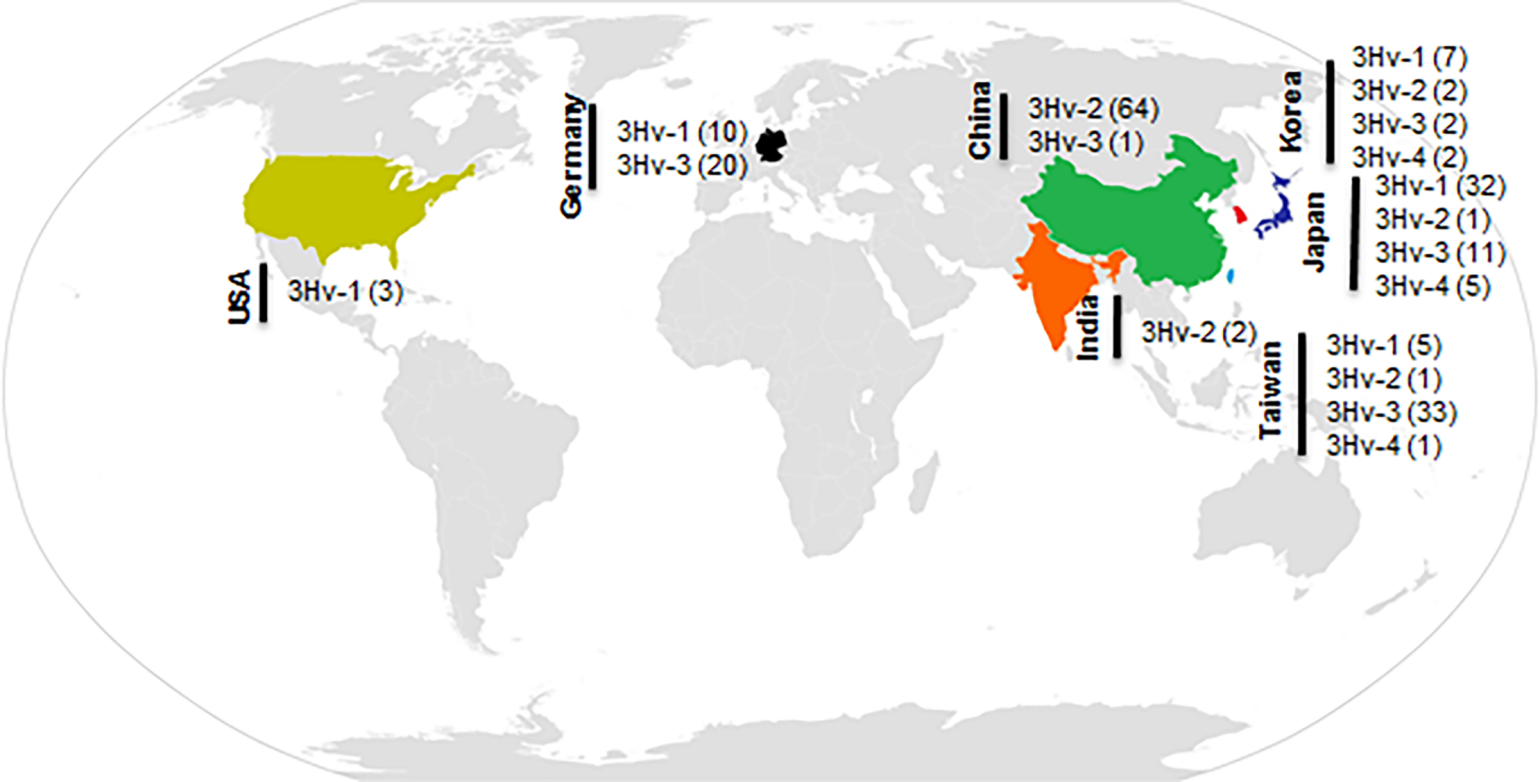
Worldwide distribution of 3Hv-1 to 3Hv-4. 3Hv-1 to 3Hv-4 comprise 80% of all variants distributed among 7 countries (China, Korea, Japan, Taiwan, Germany, the United States, and India). The values inside the brackets indicate the number of strains reported in each country.

Multiple sequence alignment revealed a total of 25 HAdV-3 hexon variants (3Hv-1 to 3Hv-25) (Fig 3). The highest number of these hexon variants was found in Korea (12 out of 25 strains, 48%), followed by China (9 out of 81 strains, 10%); Taiwan (7 out of 49 strains, 14%); Japan (5 out of 52 strains, 9%); Germany (4 out of 36 strains, 11%), the United States (1 out of 3 strains), and India (1 out of 2 strains) (Table 1). Total strains under each variant and their country based distribution are graphically depicted in Fig 4. Generally 80% of the strains (200 out of 248 strains) were included into 4 hexon variants (3Hv-1 to 3Hv-4). Among these 3Hv-2 included 28% (69) strains, followed by 3Hv-3 which comprised 27% (67) of the strains. 3Hv-1 and 3Hv-4 comprised 23% (56) and 3% (8) respectively (Fig 5).

**Table 1:** Country based distribution HAdV-3 hexon variants.

Country	Total number of strains	Hexon variants of HAdV-3
China	81	3Hv-2, 3Hv-14 to 3Hv-20 (total; 9 hexon variant)
Japan	52	3Hv-1 to 3Hv-5 (total; 5 hexon variant)
Taiwan	49	3Hv-1 to 3Hv-4, 3Hv-21 to 3Hv-23 (total;7 hexon variant)
Germany	36	3Hv-1, 3Hv-4, 3Hv-24, 3Hv-25 (total; 4 hexon variant)
Korea	25	3Hv-1 to 3Hv-4, 3Hv-6 to 3Hv-13 (total 12; hexon variant)
Unite states	3	3Hv-1 (total; 1 hexon variant) & GB strain
India	2	3Hv-2 (total; 1 hexon variant)

## Discussion

HAdV-3 is associated with sporadic infection, as well as with community and institutional outbreaks. It is evident that, for the last few years HAdV-3 respiratory infection has become a global concern, especially in the Asian countries [1,36–38]. For example, in Korea HAdV-3 comprises 23% to 54% of adenoviral respiratory tract infection while in Japan, China and Taiwan it is 33% to 50%, 70% to 78% and 2% to 87% of infection, respectively[2,6,11,12,19,24,26,36,38–42]. In the United stated HAdV-3 respiratory infection among the civilians and military population were 34.6% and 2.6% respectively [4,6]. The increased prevalence of HAdV-3 respiratory infection has been associated with severe morbidity and increased cost of medical care [43]. The hexon protein is the major antigen of HAdV and is associated with type specific immunity against the virus. In spite of the increased prevalence of HAdV-3 as a respiratory pathogen, the diversity of its hexon protein in circulating strains worldwide remains unexplored [20].

In the present study, for the first time, we analysed the hexon proteins of all the available strains of HAdV-3 from the NCBI database. We found that the HVRs of HAdV-3 strains circulating worldwide are highly heterogeneous. From our analysis it is also evident that since the first isolation of the prototype GB strain in 1953, subsequent strains have shown remarkable changes in their HVRs. Thus we conclude that the HVRs of the HAdV-3 are neither conserved nor stable. This feature enabled us to categorize the worldwide circulating strains of HAdV-3 into 25 hexon variants (3Hv-1 to 3Hv-25). Categorization into hexon variants may be valuable for clarifying immunity against the virus, the feasibility of vaccine development and the future epidemiological study of HAdV-3. It is noteworthy that this categorization is clearly different from the genomic variant (genome type) of HAdV-3 which basically represents genomic fingerprinting of HAdV-3 using multiple restriction endonuclease (RE) [12,17,18,44–47]. Although genomic variant study is useful to understand geographical distribution and global circulation of a specific strain, it does not provide information regarding immunity against HAdV-3. In this respect the proposed hexon variants can be advantageous. Furthermore the hexon variants can be useful for future epidemiological exploration of HAdV-3: incidence and prevalence of the different hexon variants, most prevalent hexon variants circulating worldwide or in a given country, geographical distribution and patterns of circulation and most prevalent currently circulating hexon variants in order to identify possible common targets for intervention. It is interesting to note that among the—25 hexon variants (3Hv1 to 3Hv25) observed in our study only 4 (3Hv-1 to 3Hv-4) comprise 80% (200 out of 248) of the circulating HAdV-3. The highest number (n = 12) of the hexon variants is reported from Korea, followed by China (n = 9), Taiwan (n = 7), Japan (n = 5), and Germany (n = 4). The number of the hexon variants of HAdV-3 is exceptionally vast in comparison with other HAdV types, as HVRs are usually conserved within the different strains of an HAdV type.

In adenoviral infection, neutralizing antibodies are directed against the epitopes located in hexon. Usually the antibody response is prolonged and provides protection against reinfection by same type when the epitopes are conserved [48,49]. Mutation in the epitope region is a mechanism of the virus to escape existing immunity. In comparison with other HAdVs types, HVRs of hexons of HAdV-3 are more prone to mutational changes as evidenced by the exceptionally large number of hexon variants in our study. The hexon epitopes are conformational; therefore AA changes in one or more HVRs may drastically affect protein folding in the antigenic regions [50]. As a result, circulating antibodies against 1 hexon variant of HAdV-3 may not be enough to protect against infection by the other hexon variants of HAdV-3. These findings explain the virological basis of the increased prevalence of global HAdV-3 infection.

A vaccine against HAdV-3 has long been in demand not only to prevent infection but also to reduce morbidity and lower the cost of medical care. The prerequisite of adenoviral vaccine development is a conserved and stable HVR. For example conserve HVRs of hexon have enabled the development of an effective vaccine against HAdV-4 and HAdV-7 [51,52]. However, the prolonged effectiveness of a vaccine is dependent on the stability of HVRs. Minor mutations in HVRs can result in antigenic drift [52]. These types of mutations have been found among different strains of HAdV-4 and HAdV-7, which helped the virus to circumvent immunity against vaccine strains and caused adult respiratory distress syndrome in vaccinated military recruits [50,51]. On the other hand, HAdV-8 is a typical example of conserved and stable HVRs of hexons [53]. In an endemic zone, existing type-specific antibodies in a population can decrease the prevalence of infection. This may be the situation that occurred in Japan; where the prevalence of epidemic keratoconjunctivitis by HAdV-8 has reduced remarkably. The vast number (25) of hexon variants of HAdV-3 indicates that development of a vaccine for global protection might be challenging. The information on the genetic variability of HAdV-3 hexon proteins provided in this study is valuable for preliminary assessments in vaccine development. However, production of antibody against currently circulating variants of HAdV-3 and performing cross-neutralization tests with other variants of HAdV-3 in cell cultures could substantiate our observation.

Studies show that HAdV-3 respiratory infection is more prevalent in Asian countries, including Japan, China, Korea, and Taiwan. As a result, research publications associated with genetic characterization using hexon sequence data of HAdV-3 strains come mainly from those countries. In comparison, there are few HAdV-3 publications from other parts of the world, such as South or Central America, Africa, Southeast Asia, or Australia. Moreover, in spite of their immense epidemiological significance, we have found that those studies do not incorporate hexon sequence data. Even then, from the available sequences from *Gen*Bank, we have succeeded in categorizing HAdV-3 strains into hexon variants. Today, rapidly advancing sequencing technologies at affordable cost have facilitated the increased number of adenovirus capsid gene sequence submissions to *Gen*Bank. We hope that in the future more HAdV-3 hexon sequences will be deposited in *Gen*Bank and that data will be included in the hexon variant study.

From our study it is apparent that the increased prevalence of HAdV-3 respiratory infection is related to the great heterogeneity of the HAdV-3 hexon protein. It is apparent that this heterogeneity may make future vaccine development highly challenging. Because HAdV-3 continues to show genetic variability, correlation between different hexon variants and their virulence would benefit the epidemiological study of HAdV-3.

## Supporting information

S1 Table HAdV-3 hexon variants data obtained from *Gen*Bank. (DOCX)

S1 Fig Above: Nucleotide sequence of the GB strain of human adenovirus type 3 (HAdV-3). The hexon gene extends from 18,798 to 19,790 bp (993 bp), a genome sequence that encodes hypervariable regions of the hexon.
Below: Predicted amino acid (AA) sequence of the hexon gene sequence mentioned above. The nucleotide sequences were translated to the predicted AA sequences using Genetyx software (Genetyx Corporation, Tokyo). This AA sequence is used as a standard for analysis.
(TIF)
