# Correction: Worldwide increased prevalence of human adenovirus type 3 (HAdV-3) respiratory infections is well correlated with heterogeneous hypervariable regions (HVRs) of hexon

**DOI:** 10.1371/journal.pone.0196263

**Published:** 2018-04-18

**Authors:** Ezazul Haque, Urmila Banik, Tahmina Monowar, Leela Anthony, Arun Kumar Adhikary

The third author's name is spelled incorrectly. The correct name is: Tahmina Monowar. The correct citation is: Haque E, Banik U, Monowar T, Anthony L, Adhikary AK (2018) Worldwide increased prevalence of human adenovirus type 3 (HAdV-3) respiratory infections is well correlated with heterogeneous hypervariable regions (HVRs) of hexon. PLoS ONE 13(3): e0194516. https://doi.org/10.1371/journal.pone.0194516
