# Supplementary material for: Worldwide increased prevalence of human adenovirus type 3 (HAdV-3) respiratory infections is well correlated with heterogeneous hypervariable regions (HVRs) of hexon
Source: PLoS One. 2018 Mar 28;13(3):e0194516. doi: 10.1371/journal.pone.0194516 (PMC5874027; doi:10.1371/journal.pone.0194516)
Supplement: S1 Table — (DOCX) [file pone.0194516.s001.docx]

**S1 Table. HAdV-3 hexon variants data obtained from *Gen*Bank**

| **No** | **Country/ Place of Origin** | **Year of collection** | **Strain** | **Year of sequence submission in GenBank** | **GenBank Accession No** | **Pubmed ID ( PMID)** | **Complete Genome (CG)**  **Complete Hexon gene (CHG)**  **Partial hexon Gene (PHG)** | **Hexon**  **Variant of HAdV-3 (3Hv)** |
| --- | --- | --- | --- | --- | --- | --- | --- | --- |
| 1 | Guangzhou | 2005 | ad01 | 2005 | AY878716 | - | CHG | 3Hv-14 |
| 2 | Beijing | 2011 | BJ02 | 2014 | KM458623 | [25816320](https://www.ncbi.nlm.nih.gov/pubmed/25816320) | CHG | 3Hv-2 |
| 3 | Beijing | 2012 | BJ03 | 2014 | KM458624 | [25816320](https://www.ncbi.nlm.nih.gov/pubmed/25816320) | CHG | 3Hv-2 |
| 4 | Beijing | 2013 | BJ19 | 2014 | KM458630 | [25816320](https://www.ncbi.nlm.nih.gov/pubmed/25816320) | CHG | 3Hv-2 |
| 5 | Chongqing | 2010 | CQ1036 | 2012 | JQ412603 | - | PHG | 3Hv-2 |
| 6 | Chongqing | 2010 | CQ1169 | 2012 | JQ412604 | - | PHG | 3Hv-20 |
| 7 | Chongqing | 2010 | CQ1209 | 2012 | JQ412605 | - | PHG | 3Hv-20 |
| 8 | Chongqing | 2010 | CQ1242 | 2012 | JQ412606 | - | PHG | 3Hv-2 |
| 9 | Chongqing | 2011 | CQ1256 | 2012 | JQ412607 | - | PHG | 3Hv-20 |
| 10 | Chongqing | 2011 | CQ1265 | 2012 | JQ412608 | - | PHG | 3Hv-20 |
| 11 | Chongqing | 2011 | CQ1278 | 2012 | JQ412609 | - | PHG | 3Hv-20 |
| 12 | Chongqing | 2011 | CQ1289 | 2012 | JQ412610 | - | PHG | 3Hv-2 |
| 13 | Chongqing | 2011 | CQ1300 | 2012 | JQ412611 | - | PHG | 3Hv-2 |
| 14 | Chongqing | 2011 | CQ1325 | 2012 | JQ412612 | - | PHG | 3Hv-2 |
| 15 | Chongqing | 2011 | CQ1351 | 2012 | JQ412613 | - | PHG | 3Hv-2 |
| 16 | Chongqing | 2011 | CQ1623 | 2012 | JQ412614 | - | PHG | 3Hv-2 |
| 17 | Chongqing | 2011 | CQ1691 | 2012 | JQ412615 | - | PHG | 3Hv-2 |
| 18 | Chongqing | 2009 | CQ573 | 2012 | JQ412616 | - | PHG | 3Hv-20 |
| 19 | Chongqing | 2009 | CQ614 | 2012 | JQ412617 | - | PHG | 3Hv-20 |
| 20 | Chongqing | 2009 | CQ641 | 2012 | JQ412618 | - | PHG | 3Hv-2 |
| 21 | Chongqing | 2009 | CQ690 | 2012 | JQ412619 | - | PHG | 3Hv-2 |
| 22 | Chongqing | 2009 | CQ692 | 2012 | JQ412620 | - | PHG | 3Hv-2 |
| 23 | Chongqing | 2009 | CQ709 | 2012 | JQ412621 | - | PHG | 3Hv-2 |
| 24 | Chongqing | 2009 | CQ764 | 2012 | JQ412622 | - | PHG | 3Hv-2 |
| 25 | Chongqing | 2010 | CQ800 | 2012 | JQ412623 | - | PHG | 3Hv-16 |
| 26 | Chongqing | 2010 | CQ806 | 2012 | JQ412624 | - | PHG | 3Hv-2 |
| 27 | Chongqing | 2010 | CQ932 | 2012 | JQ412625 | - | PHG | 3Hv-17 |
| 28 | Chongqing | 2009 | CQ97 | 2012 | JQ412626 | - | PHG | 3Hv-20 |
| 29 | Guangzhou | 2005 | Guangzhou01 | 2006 | DQ099432 | [16690917](https://www.ncbi.nlm.nih.gov/pubmed/16690917) | CG | 3Hv-2 |
| 30 | Guangzhou | 2004 | Guangzhou02 | 2006 | DQ105654 | [16690917](https://www.ncbi.nlm.nih.gov/pubmed/16690917) | CG | 3Hv-3 |
| 31 | Guangzhou | 2012 | GZ_49_2012 | 2015 | KR090744 | [27171486](https://www.ncbi.nlm.nih.gov/pubmed/27171486) | PHG | 3Hv-2 |
| 32 | Guangzhou | 2012 | GZ_050_2012 | 2015 | KR090745 | [27171486](https://www.ncbi.nlm.nih.gov/pubmed/27171486) | PHG | 3Hv-2 |
| 33 | Guangzhou | 2012 | GZ_057_2012 | 2015 | KR090746 | [27171486](https://www.ncbi.nlm.nih.gov/pubmed/27171486) | PHG | 3Hv-2 |
| 34 | Guangzhou | 2012 | GZ_058_2012 | 2015 | KR090747 | [27171486](https://www.ncbi.nlm.nih.gov/pubmed/27171486) | PHG | 3Hv-2 |
| 35 | Guangzhou | 2012 | GZ_073_2012 | 2015 | KR090748 | [27171486](https://www.ncbi.nlm.nih.gov/pubmed/27171486) | PHG | 3Hv-2 |
| 36 | Guangzhou | 2012 | GZ_75_2012 | 2015 | KR090750 | [27171486](https://www.ncbi.nlm.nih.gov/pubmed/27171486) | PHG | 3Hv-2 |
| 37 | Guangzhou | 2012 | GZ_076_2012 | 2015 | KR090751 | [27171486](https://www.ncbi.nlm.nih.gov/pubmed/27171486) | PHG | 3Hv-2 |
| 38 | Guangzhou | 2012 | GZ_087_2012 | 2015 | KR090754 | [27171486](https://www.ncbi.nlm.nih.gov/pubmed/27171486) | PHG | 3Hv-2 |
| 39 | Guangzhou | 2012 | GZ_95_2012 | 2015 | KR090756 | [27171486](https://www.ncbi.nlm.nih.gov/pubmed/27171486) | PHG | 3Hv-2 |
| 40 | Guangzhou | 2012 | GZ_121_2012 | 2015 | KR090757 | [27171486](https://www.ncbi.nlm.nih.gov/pubmed/27171486) | PHG | 3Hv-2 |
| 41 | Guangzhou | 2013 | GZ_172_2013 | 2015 | KR090758 | [27171486](https://www.ncbi.nlm.nih.gov/pubmed/27171486) | PHG | 3Hv-2 |
| 42 | Guangzhou | 2013 | GZ_174_2013 | 2015 | KR090759 | [27171486](https://www.ncbi.nlm.nih.gov/pubmed/27171486) | PHG | 3Hv-2 |
| 43 | Guangzhou | 2013 | GZ_177_2013 | 2015 | KR090760 | [27171486](https://www.ncbi.nlm.nih.gov/pubmed/27171486) | PHG | 3Hv-2 |
| 44 | Guangzhou | 2013 | GZ_188_2013 | 2015 | KR090761 | [27171486](https://www.ncbi.nlm.nih.gov/pubmed/27171486) | PHG | 3Hv-2 |
| 45 | Guangzhou | 2013 | GZ_190_2013 | 2015 | KR090763 | [27171486](https://www.ncbi.nlm.nih.gov/pubmed/27171486) | PHG | 3Hv-2 |
| 46 | Guangzhou | 2013 | GZ_192_2013 | 2015 | KR090764 | [27171486](https://www.ncbi.nlm.nih.gov/pubmed/27171486) | PHG | 3Hv-2 |
| 47 | Guangzhou | 2013 | GZ_196_2013 | 2015 | KR090766 | [27171486](https://www.ncbi.nlm.nih.gov/pubmed/27171486) | PHG | 3Hv-2 |
| 48 | Guangzhou | 2013 | GZ_198_2013 | 2015 | KR090767 | [27171486](https://www.ncbi.nlm.nih.gov/pubmed/27171486) | PHG | 3Hv-2 |
| 49 | Guangzhou | 2013 | GZ_208_2013 | 2015 | KR090769 | [27171486](https://www.ncbi.nlm.nih.gov/pubmed/27171486) | PHG | 3Hv-2 |
| 50 | Guangzhou | 2013 | GZ_212_2013 | 2015 | KR090770 | [27171486](https://www.ncbi.nlm.nih.gov/pubmed/27171486) | PHG | 3Hv-2 |
| 51 | Guangzhou | 2013 | GZ_222_2013 | 2015 | KR090772 | [27171486](https://www.ncbi.nlm.nih.gov/pubmed/27171486) | PHG | 3Hv-2 |
| 52 | Guangzhou | 2013 | GZ_225_2013 | 2015 | KR090774 | [27171486](https://www.ncbi.nlm.nih.gov/pubmed/27171486) | PHG | 3Hv-2 |
| 53 | Guangzhou | 2013 | GZ_226_2013 | 2015 | KR090775 | [27171486](https://www.ncbi.nlm.nih.gov/pubmed/27171486) | PHG | 3Hv-2 |
| 54 | Guangzhou | 2013 | GZ_229_2013 | 2015 | KR090776 | [27171486](https://www.ncbi.nlm.nih.gov/pubmed/27171486) | PHG | 3Hv-2 |
| 55 | Guangzhou | 2013 | GZ_235_2013 | 2015 | KR090777 | [27171486](https://www.ncbi.nlm.nih.gov/pubmed/27171486) | PHG | 3Hv-2 |
| 56 | Guangzhou | 2013 | GZ_239_2013 | 2015 | KR090779 | [27171486](https://www.ncbi.nlm.nih.gov/pubmed/27171486) | PHG | 3Hv-19 |
| 57 | Guangzhou | 2013 | GZ_240_2013 | 2015 | KR090780 | [27171486](https://www.ncbi.nlm.nih.gov/pubmed/27171486) | PHG | 3Hv-2 |
| 58 | Guangzhou | 2013 | GZ_249_2013 | 2015 | KR090783 | [27171486](https://www.ncbi.nlm.nih.gov/pubmed/27171486) | PHG | 3Hv-19 |
| 59 | Guangzhou | 2013 | GZ_256_2013 | 2015 | KR090784 | [27171486](https://www.ncbi.nlm.nih.gov/pubmed/27171486) | PHG | 3Hv-2 |
| 60 | Guangzhou | 2013 | GZ_259_2013 | 2015 | KR090785 | [27171486](https://www.ncbi.nlm.nih.gov/pubmed/27171486) | PHG | 3Hv-2 |
| 61 | Guangzhou | 2013 | GZ_260_2013 | 2015 | KR090787 | [27171486](https://www.ncbi.nlm.nih.gov/pubmed/27171486) | PHG | 3Hv-2 |
| 62 | Guangzhou | 2013 | GZ_05273_2013 | 2015 | KR090789 | [27171486](https://www.ncbi.nlm.nih.gov/pubmed/27171486) | PHG | 3Hv-2 |
| 63 | Guangzhou | 2013 | GZ_04803_2013 | 2015 | KR090794 | [27171486](https://www.ncbi.nlm.nih.gov/pubmed/27171486) | PHG | 3Hv-2 |
| 64 | Guangzhou | 2013 | GZ_05265_2013 | 2015 | KR090795 | [27171486](https://www.ncbi.nlm.nih.gov/pubmed/27171486) | PHG | 3Hv-2 |
| 65 | Guangzhou | 2013 | GZ_07803_2013 | 2015 | KR090796 | [27171486](https://www.ncbi.nlm.nih.gov/pubmed/27171486) | PHG | 3Hv-20 |
| 66 | Guangzhou | 2013 | GZ_13803_2013 | 2015 | KR090799 | [27171486](https://www.ncbi.nlm.nih.gov/pubmed/27171486) | PHG | 3Hv-2 |
| 67 | Guangzhou | 2013 | GZ_30803_2013 | 2015 | KR090800 | [27171486](https://www.ncbi.nlm.nih.gov/pubmed/27171486) | PHG | 3Hv-2 |
| 68 | Guangzhou | 2011 | GZ_28_2011 | 2015 | KR090801 | [27171486](https://www.ncbi.nlm.nih.gov/pubmed/27171486) | PHG | 3Hv-2 |
| 69 | Guangzhou | 2011 | GZ_30_2011 | 2015 | KR090802 | [27171486](https://www.ncbi.nlm.nih.gov/pubmed/27171486) | PHG | 3Hv-2 |
| 70 | Guangzhou | 2011 | GZ_31_2011 | 2015 | KR090803 | [27171486](https://www.ncbi.nlm.nih.gov/pubmed/27171486) | PHG | 3Hv-2 |
| 71 | Guangzhou | 2011 | GZ_36_2011 | 2015 | KR090804 | [27171486](https://www.ncbi.nlm.nih.gov/pubmed/27171486) | PHG | 3Hv-2 |
| 72 | Guangzhou | 2011 | GZ_38_2011 | 2015 | KR090806 | [27171486](https://www.ncbi.nlm.nih.gov/pubmed/27171486) | PHG | 3Hv-2 |
| 73 | Guangzhou | 2011 | GZ_43_2011 | 2015 | KR090808 | [27171486](https://www.ncbi.nlm.nih.gov/pubmed/27171486) | PHG | 3Hv-2 |
| 74 | Guangzhou | 2011 | GZ_45_2011 | 2015 | KR090809 | [27171486](https://www.ncbi.nlm.nih.gov/pubmed/27171486) | PHG | 3Hv-2 |
| 75 | Guangzhou | 2011 | GZ_55_2011 | 2015 | KR090811 | [27171486](https://www.ncbi.nlm.nih.gov/pubmed/27171486) | PHG | 3Hv-2 |
| 76 | Guangzhou | 2011 | GZ_58_2011 | 2015 | KR090813 | [27171486](https://www.ncbi.nlm.nih.gov/pubmed/27171486) | PHG | 3Hv-20 |
| 77 | Guangzhou | 2011 | GZ_66_2011 | 2015 | KR090814 | [27171486](https://www.ncbi.nlm.nih.gov/pubmed/27171486) | PHG | 3Hv-2 |
| 78 | Guangzhou | 2011 | GZ_77_2011 | 2015 | KR090815 | [27171486](https://www.ncbi.nlm.nih.gov/pubmed/27171486) | PHG | 3Hv-2 |
| 79 | Guangzhou | 2011 | GZ_95_2011 | 2015 | KR090817 | [27171486](https://www.ncbi.nlm.nih.gov/pubmed/27171486) | PHG | 3Hv-2 |
| 80 | Guangzhou | 2011 | GZ_106_2011 | 2015 | KR090818 | [27171486](https://www.ncbi.nlm.nih.gov/pubmed/27171486) | PHG | 3Hv-2 |
| 81 | Guangzhou | 2011 | GZ13 | 2012 | JQ764730 | - | CHG | 3Hv-18 |
| 82 | Jiangsu | - | Jiangsu04-1 | 2005 | DQ177310 | - | PHG | 3Hv-15 |
| **1** | **GERMANY** | 2002 | RKI-1332/02 | 2008 | EU867456 | [20149723](https://www.ncbi.nlm.nih.gov/pubmed/20149723) | PHG | 3Hv-3 |
| 2 | Germany | 2005 | RKI-0654/06 | 2008 | EU867473 | [20149723](https://www.ncbi.nlm.nih.gov/pubmed/20149723) | PHG | 3Hv-1 |
| 3 | Germany | 2006 | RKI-1206/06 | 2008 | EU867475 | [20149723](https://www.ncbi.nlm.nih.gov/pubmed/20149723) | PHG | 3Hv-3 |
| 4 | Germany | 2006 | RKI-3832/06 | 2008 | EU867477 | [20149723](https://www.ncbi.nlm.nih.gov/pubmed/20149723) | PHG | 3Hv-1 |
| 5 | Germany | 2007 | RKI-3723/07 | 2008 | EU867487 | [20149723](https://www.ncbi.nlm.nih.gov/pubmed/20149723) | PHG | 3Hv-3 |
| 6 | Germany | 2007 | RKI-3825/07 | 2008 | EU867488 | [20149723](https://www.ncbi.nlm.nih.gov/pubmed/20149723) | PHG | 3Hv-24 |
| 7 | Germany | 2007 | RKI-4011/07 | 2008 | EU867489 | [20149723](https://www.ncbi.nlm.nih.gov/pubmed/20149723) | PHG | 3Hv-1 |
| 8 | Germany | 2007 | RKI-4145/07 | 2008 | EU867490 | [20149723](https://www.ncbi.nlm.nih.gov/pubmed/20149723) | PHG | 3Hv-3 |
| 9 | Germany | 2007 | RKI-4289/07 | 2008 | EU867491 | [20149723](https://www.ncbi.nlm.nih.gov/pubmed/20149723) | PHG | 3Hv-3 |
| 10 | Germany | 2001 | RKI-0273/02 | 2009 | FJ943583 | [20149723](https://www.ncbi.nlm.nih.gov/pubmed/20149723) | PHG | 3Hv-3 |
| 11 | Germany | 2001 | RKI-0602/02 | 2009 | FJ943586 | [20149723](https://www.ncbi.nlm.nih.gov/pubmed/20149723) | PHG | 3Hv-1 |
| 12 | Germany | 2001 | RKI-0622/02 | 2009 | FJ943587 | [20149723](https://www.ncbi.nlm.nih.gov/pubmed/20149723) | PHG | 3Hv-25 |
| 13 | Germany | 2002 | RKI-0950/02 | 2009 | FJ943590 | [20149723](https://www.ncbi.nlm.nih.gov/pubmed/20149723) | PHG | 3Hv-1 |
| 14 | Germany | 2002 | RKI-0954/02 | 2009 | FJ943591 | [20149723](https://www.ncbi.nlm.nih.gov/pubmed/20149723) | PHG | 3Hv-1 |
| 15 | Germany | 2002 | RKI-1825/02 | 2009 | FJ943593 | [20149723](https://www.ncbi.nlm.nih.gov/pubmed/20149723) | PHG | 3Hv-3 |
| 16 | Germany | 2002 | RKI-2043/02 | 2009 | FJ943594 | [20149723](https://www.ncbi.nlm.nih.gov/pubmed/20149723) | PHG | 3Hv-1 |
| 17 | Germany | 2003 | RKI-2548/03 | 2009 | FJ943598 | [20149723](https://www.ncbi.nlm.nih.gov/pubmed/20149723) | PHG | 3Hv-3 |
| 18 | Germany | 2006 | RKI-0549/07 | 2009 | FJ943600 | [20149723](https://www.ncbi.nlm.nih.gov/pubmed/20149723) | PHG | 3Hv-25 |
| 19 | Germany | 2007 | RKI-0618/07 | 2009 | FJ943601 | [20149723](https://www.ncbi.nlm.nih.gov/pubmed/20149723) | PHG | 3Hv-25 |
| 20 | Germany | 2007 | RKI-0918/07 | 2009 | FJ943604 | [20149723](https://www.ncbi.nlm.nih.gov/pubmed/20149723) | PHG | 3Hv-3 |
| 21 | Germany | 2007 | RKI-0973/07 | 2009 | FJ943606 | [20149723](https://www.ncbi.nlm.nih.gov/pubmed/20149723) | PHG | 3Hv-25 |
| 22 | Germany | 2007 | RKI-1093/07 | 2009 | FJ943607 | [20149723](https://www.ncbi.nlm.nih.gov/pubmed/20149723) | PHG | 3Hv-3 |
| 23 | Germany | 2007 | RKI-1395/07 | 2009 | FJ943612 | [20149723](https://www.ncbi.nlm.nih.gov/pubmed/20149723) | PHG | 3Hv-1 |
| 24 | Germany | 2007 | RKI-2135/07 | 2009 | FJ943618 | [20149723](https://www.ncbi.nlm.nih.gov/pubmed/20149723) | PHG | 3Hv-3 |
| 25 | Germany | 2007 | RKI-2183/07 | 2009 | FJ943619 | [20149723](https://www.ncbi.nlm.nih.gov/pubmed/20149723) | PHG | 3Hv-1 |
| 26 | Germany | 2007 | RKI-2350/07 | 2009 | FJ943620 | [20149723](https://www.ncbi.nlm.nih.gov/pubmed/20149723) | PHG | 3Hv-3 |
| 27 | Germany | 2007 | RKI-2808/07 | 2009 | FJ943622 | [20149723](https://www.ncbi.nlm.nih.gov/pubmed/20149723) | PHG | 3Hv-3 |
| 28 | Germany | 2007 | RKI-3110/07 | 2009 | FJ943624 | [20149723](https://www.ncbi.nlm.nih.gov/pubmed/20149723) | PHG | 3Hv-1 |
| 29 | Germany | 2007 | RKI-3231/07 | 2009 | FJ943625 | [20149723](https://www.ncbi.nlm.nih.gov/pubmed/20149723) | PHG | 3Hv-3 |
| 30 | Germany | 2007 | RKI-3263/07 | 2009 | FJ943626 | [20149723](https://www.ncbi.nlm.nih.gov/pubmed/20149723) | PHG | 3Hv-3 |
| 31 | Germany | 2007 | RKI-3576/07 | 2009 | FJ943629 | [20149723](https://www.ncbi.nlm.nih.gov/pubmed/20149723) | PHG | 3Hv-3 |
| 32 | Germany | 2007 | RKI-3591/07 | 2009 | FJ943630 | [20149723](https://www.ncbi.nlm.nih.gov/pubmed/20149723) | PHG | 3Hv-3 |
| 33 | Germany | 2007 | RKI-3646/07 | 2009 | FJ943632 | [20149723](https://www.ncbi.nlm.nih.gov/pubmed/20149723) | PHG | 3Hv-3 |
| 34 | Germany | 2007 | RKI-3827/07 | 2009 | FJ943634 | [20149723](https://www.ncbi.nlm.nih.gov/pubmed/20149723) | PHG | 3Hv-3 |
| 35 | Germany | 2007 | RKI-3986/07 | 2009 | FJ943636 | [20149723](https://www.ncbi.nlm.nih.gov/pubmed/20149723) | PHG | 3Hv-25 |
| 36 | Germany | 2007 | RKI-4263/07 | 2009 | FJ943637 | [20149723](https://www.ncbi.nlm.nih.gov/pubmed/20149723) | PHG | 3Hv-3 |
| **1** | **INDIA** | 2011 | human/IND/MEEI_00057/2011/3 | 2013 | KF268210 | - | CG | 3Hv-2 |
| 2 | India | 2011 | human/IND/MEEI_00062/2011/3[P7H3F3 | 2013 | KF268212 | - | CG | 3Hv-2 |
| **1** | **JAPAN** | NA | y | 2008 | AB375857 | [18362407](https://www.ncbi.nlm.nih.gov/pubmed/18362407) | PHG | 3Hv-5 |
| 2 | Yamagata | NA | Ad3P | 2001 | AB067658 | [19038295](https://www.ncbi.nlm.nih.gov/pubmed/19038295) | PHG | GB |
| 3 | Yamagata | NA | Ad3a | 2001 | AB067659 | [19038295](https://www.ncbi.nlm.nih.gov/pubmed/19038295) | PHG | 3Hv-1 |
| 4 | Yamagata | 1986 | Ad3Y86-1555 | 2001 | AB067660 | - | PHG | 3Hv-1 |
| 5 | Yamagata | 1986 | Ad3Y86-2866 | 2001 | AB067661 | - | PHG | 3Hv-1 |
| 6 | Yamagata | 1987 | Ad3Y87-2170 | 2001 | AB067662 | - | PHG | 3Hv-1 |
| 7 | Yamagata | 1987 | Ad3Y87-3388 | 2001 | AB067663 | - | PHG | 3Hv-1 |
| 8 | Yamagata | 1989 | Ad3Y89-702 | 2001 | AB067664 | [19038295](https://www.ncbi.nlm.nih.gov/pubmed/19038295) | PHG | 3Hv-1 |
| 9 | Yamagata | 1989 | Ad3Y89-2632 | 2001 | AB067665 | [19038295](https://www.ncbi.nlm.nih.gov/pubmed/19038295) | PHG | 3Hv-1 |
| 10 | Yamagata | 1989 | Ad3Y89-4067 | 2001 | AB067666 | [19038295](https://www.ncbi.nlm.nih.gov/pubmed/19038295) | PHG | 3Hv-1 |
| 11 | Yamagata | 1990 | Ad3Y90-4578 | 2001 | AB067667 | [19038295](https://www.ncbi.nlm.nih.gov/pubmed/19038295) | PHG | 3Hv-1 |
| 12 | Yamagata | 1992 | Ad3Y92-189 | 2001 | AB067668 | [19038295](https://www.ncbi.nlm.nih.gov/pubmed/19038295) | PHG | 3Hv-1 |
| 13 | Yamagata | 1994 | Ad3Y94-2330 | 2001 | AB067669 | [19038295](https://www.ncbi.nlm.nih.gov/pubmed/19038295) | PHG | 3Hv-1 |
| 14 | Yamagata | 1995 | Ad3Y95-2087 | 2001 | AB067670 | [19038295](https://www.ncbi.nlm.nih.gov/pubmed/19038295) | PHG | 3Hv-1 |
| 15 | Yamagata | 1996 | Ad3Y96-224 | 2001 | AB067671 | [19038295](https://www.ncbi.nlm.nih.gov/pubmed/19038295) | PHG | 3Hv-1 |
| 16 | Yamagata | 1998 | Ad3Y98-9 | 2001 | AB067672 | [19038295](https://www.ncbi.nlm.nih.gov/pubmed/19038295) | PHG | 3Hv-2 |
| 17 | Yamagata | 1993 | Ad3A326-G93 | 2001 | AB067673 | - | PHG | 3Hv-1 |
| 18 | Yamagata | 1993 | Ad3A724-G93 | 2001 | AB067674 | - | PHG | 3Hv-1 |
| 19 | Yamagata | 1995 | Ad3A586-G95 | 2001 | AB067675 | - | PHG | 3Hv-1 |
| 20 | Yamagata | 1996 | Ad3A9-C96 | 2001 | AB067676 | - | PHG | 3Hv-1 |
| 21 | Yamagata | 1996 | Ad3A432-G96 | 2001 | AB067677 | - | PHG | 3Hv-1 |
| 22 | Yamagata | 1988 | Ad3Y88-507 | 2007 | AB366402 | [19038295](https://www.ncbi.nlm.nih.gov/pubmed/19038295) | PHG | 3Hv-1 |
| 23 | Yamagata | 1988 | Ad3Y88-608 | 2007 | AB366403 | [19038295](https://www.ncbi.nlm.nih.gov/pubmed/19038295) | PHG | 3Hv-1 |
| 24 | Yamagata | 1988 | Ad3Y88-5309 | 2007 | AB366404 | [19038295](https://www.ncbi.nlm.nih.gov/pubmed/19038295) | PHG | 3Hv-1 |
| 25 | Yamagata | 1990 | Ad3Y90-3080 | 2007 | AB366405 | [19038295](https://www.ncbi.nlm.nih.gov/pubmed/19038295) | PHG | 3Hv-1 |
| 26 | Yamagata | 1991 | Ad3Y91-2489 | 2007 | AB366406 | [19038295](https://www.ncbi.nlm.nih.gov/pubmed/19038295) | PHG | 3Hv-1 |
| 27 | Yamagata | 1991 | Ad3Y91-3246 | 2007 | AB366407 | [19038295](https://www.ncbi.nlm.nih.gov/pubmed/19038295) | PHG | 3Hv-1 |
| 28 | Yamagata | 1992 | Ad3Y92-884 | 2007 | AB366408 | [19038295](https://www.ncbi.nlm.nih.gov/pubmed/19038295) | PHG | 3Hv-1 |
| 29 | Yamagata | 1993 | Ad3Y93-58 | 2007 | AB366409 | [19038295](https://www.ncbi.nlm.nih.gov/pubmed/19038295) | PHG | 3Hv-1 |
| 30 | Yamagata | 1993 | Ad3Y93-930 | 2007 | AB366410 | [19038295](https://www.ncbi.nlm.nih.gov/pubmed/19038295) | PHG | 3Hv-1 |
| 31 | Yamagata | 1994 | Ad3Y94-2591 | 2007 | AB366411 | [19038295](https://www.ncbi.nlm.nih.gov/pubmed/19038295) | PHG | 3Hv-1 |
| 32 | Yamagata | 1995 | Ad3Y95-171 | 2007 | AB366412 | [19038295](https://www.ncbi.nlm.nih.gov/pubmed/19038295) | PHG | 3Hv-1 |
| 33 | Yamagata | 1997 | Ad3Y97-1022 | 2007 | AB366413 | [19038295](https://www.ncbi.nlm.nih.gov/pubmed/19038295) | PHG | 3Hv-1 |
| 34 | Yamagata | 1997 | Ad3Y97-1721 | 2007 | AB366414 | [19038295](https://www.ncbi.nlm.nih.gov/pubmed/19038295) | PHG | 3Hv-3 |
| 35 | Yamagata | 1998 | Ad3Y98-681 | 2007 | AB366415 | [19038295](https://www.ncbi.nlm.nih.gov/pubmed/19038295) | PHG | 3Hv-3 |
| 36 | Yamagata | 1999 | 989-Yamagata-99 | 2007 | AB366416 | [19038295](https://www.ncbi.nlm.nih.gov/pubmed/19038295) | PHG | 3Hv-1 |
| 37 | Yamagata | 2000 | 1091-Yamagata-00 | 2007 | AB366417 | [19038295](https://www.ncbi.nlm.nih.gov/pubmed/19038295) | PHG | 3Hv-4 |
| 38 | Yamagata | 2000 | 1115-Yamagata-00 | 2007 | AB366418 | [19038295](https://www.ncbi.nlm.nih.gov/pubmed/19038295) | PHG | 3Hv-4 |
| 39 | Yamagata | 2001 | 98-Yamagata-01 | 2007 | AB366419 | [19038295](https://www.ncbi.nlm.nih.gov/pubmed/19038295) | PHG | 3Hv-4 |
| 40 | Yamagata | 2001 | 1370-Yamagata-01 | 2007 | AB366420 | [19038295](https://www.ncbi.nlm.nih.gov/pubmed/19038295) | PHG | 3Hv-3 |
| 41 | Yamagata | 2002 | 2191-Yamagata-02 | 2007 | AB366421 | [19038295](https://www.ncbi.nlm.nih.gov/pubmed/19038295) | PHG | 3Hv-5 |
| 42 | Yamagata | 2003 | 1874-Yamagata-03 | 2007 | AB366422 | [19038295](https://www.ncbi.nlm.nih.gov/pubmed/19038295) | PHG | 3Hv-3 |
| 43 | Yamagata | 2003 | 3098-Yamagata-03 | 2007 | AB366423 | [19038295](https://www.ncbi.nlm.nih.gov/pubmed/19038295) | PHG | 3Hv-3 |
| 44 | Yamagata | 2004 | 2070-Yamagata-04 | 2007 | AB366424 | [19038295](https://www.ncbi.nlm.nih.gov/pubmed/19038295) | PHG | 3Hv-3 |
| 45 | Yamagata | 2004 | 2133-Yamagata-04 | 2007 | AB366425 | [19038295](https://www.ncbi.nlm.nih.gov/pubmed/19038295) | PHG | 3Hv-3 |
| 46 | Yamagata | 2005 | 246-Yamagata-05 | 2007 | AB366426 | [19038295](https://www.ncbi.nlm.nih.gov/pubmed/19038295) | PHG | 3Hv-3 |
| 47 | Yamagata | 2005 | 571-Yamagata-05 | 2007 | AB366427 | [19038295](https://www.ncbi.nlm.nih.gov/pubmed/19038295) | PHG | 3Hv-3 |
| 48 | Yamagata | 2006 | 1382-Yamagata-06 | 2007 | AB366428 | [19038295](https://www.ncbi.nlm.nih.gov/pubmed/19038295) | PHG | 3Hv-3 |
| 49 | Yamagata | 2006 | 2435-Yamagata-06 | 2007 | AB366429 | [19038295](https://www.ncbi.nlm.nih.gov/pubmed/19038295) | PHG | 3Hv-3 |
| 50 | Yamagata | 2007 | 57-Yamagata-07 | 2007 | AB366430 | [19038295](https://www.ncbi.nlm.nih.gov/pubmed/19038295) | PHG | 3Hv-4 |
| 51 | Yamagata | 2007 | 285-Yamagata-07 | 2007 | AB366431 | [19038295](https://www.ncbi.nlm.nih.gov/pubmed/19038295) | PHG | 3Hv-4 |
| 52 | Yamagata | - | Ad3C | 2007 | AB366432 | [19038295](https://www.ncbi.nlm.nih.gov/pubmed/19038295) | PHG | 3Hv-1 |
| **1** | **KOREA** | 1991 | 91_353 | 2004 | AY854173 | [16419117](https://www.ncbi.nlm.nih.gov/pubmed/16419117) | CHG | 3Hv-2 |
| 2 | Korea | 1992 | 92_159 | 2004 | AY854174 | [16419117](https://www.ncbi.nlm.nih.gov/pubmed/16419117) | CHG | 3Hv-1 |
| 3 | Korea | 1992 | 92_165 | 2004 | AY854175 | [16419117](https://www.ncbi.nlm.nih.gov/pubmed/16419117) | CHG | 3Hv-2 |
| 4 | Korea | 1998 | 98_494 | 2004 | AY854176 | [16419117](https://www.ncbi.nlm.nih.gov/pubmed/16419117) | CHG | 3Hv-3 |
| 5 | Korea | 1998 | 98_640 | 2004 | AY854177 | [16419117](https://www.ncbi.nlm.nih.gov/pubmed/16419117) | CHG | 3Hv-1 |
| 6 | Korea | 1999 | 99_453 | 2004 | AY854178 | [16419117](https://www.ncbi.nlm.nih.gov/pubmed/16419117) | CHG | 3Hv-4 |
| 7 | Korea | 1992 | 92-393 | 2004 | AY854179 | [16419117](https://www.ncbi.nlm.nih.gov/pubmed/16419117) | CHG | 3Hv-1 |
| 8 | Korea | 1998 | 98_500 | 2004 | AY854180 | [16419117](https://www.ncbi.nlm.nih.gov/pubmed/16419117) | CHG | 3Hv-4 |
| 9 | Korea | 1998 | KNIH Ad98/1 | 2002 | AF542104 | - | CHG | 3Hv-6 |
| 10 | Korea | 1999 | KNIH Ad99/1 | 2002 | AF542105 | - | CHG | 3Hv-1 |
| 11 | Korea | 1999 | KNIH Ad99/2 | 2002 | AF542106 | - | CHG | 3Hv-1 |
| 12 | Korea | 1999 | KNIH Ad99/3 | 2002 | AF542107 | - | CHG | 3Hv-1 |
| 13 | Korea | 1999 | KNIH Ad99/4 | 2002 | AF542108 | - | CHG | 3Hv-7 |
| 14 | Korea | 1999 | KNIH Ad99/6 | 2002 | AF542110 | - | CHG | 3Hv-8 |
| 15 | Korea | 1999 | KNIH Ad99/7 | 2002 | AF542111 | - | CHG | 3Hv-9 |
| 16 | Korea | 1999 | KNIH Ad99/8 | 2002 | AF542112 | - | CHG | 3Hv-9 |
| 17 | Korea | 1999 | KNIH Ad99/9 | 2002 | AF542113 | - | CHG | 3Hv-6 |
| 18 | Korea | 1999 | KNIH Ad99/10 | 2002 | AF542114 | - | CHG | 3Hv-1 |
| 19 | Korea | 1999 | KNIH Ad99/11 | 2002 | AF542115 | - | CHG | 3Hv-10 |
| 20 | Korea | 1999 | KNIH Ad99/13 | 2002 | AF542117 | - | CHG | 3Hv-11 |
| 21 | Korea | 2000 | KNIH Ad00/10 | 2002 | AF542123 | - | CHG | 3Hv-12 |
| 22 | Korea | 2000 | KNIH Ad00/16 | 2002 | AF542125 | - | CHG | 3Hv-13 |
| 23 | Korea | 2000 | KNIH Ad00/17 | 2002 | AF542126 | - | CHG | 3Hv-3 |
| 24 | Korea | 2000 | KNIH Ad00/18 | 2002 | AF542127 | - | CHG | 3Hv-13 |
| 25 | Korea | 2000 | KNIH Ad00/7 | 2002 | AF542129 | - | CHG | 3Hv-12 |
| **1** | **TAIWAN** | 2002 | N3706/TW/02 | 2013 | KC570875 | [24073254](https://www.ncbi.nlm.nih.gov/pubmed/24073254) | PHG | 3Hv-3 |
| 2 | Taiwan | 2005 | N725/TW/05 | 2013 | KC570876 | [24073254](https://www.ncbi.nlm.nih.gov/pubmed/24073254) | PHG | 3Hv-3 |
| 3 | Taiwan | 2009 | N8630/TW/09-2 | 2013 | KC570877 | [24073254](https://www.ncbi.nlm.nih.gov/pubmed/24073254) | PHG | 3Hv-3 |
| 4 | Taiwan | 2003 | N78/TW/03 | 2013 | KC570906 | [24073254](https://www.ncbi.nlm.nih.gov/pubmed/24073254) | PHG | 3Hv-3 |
| 5 | Taiwan | 2011 | 2902/Taiwan/2011 | 2013 | KC456083 | - | CHG | 3Hv-3 |
| 6 | Taiwan | 2011 | 0198/Taiwan/2011 | 2013 | KC456084 | - | CHG | 3Hv-3 |
| 7 | Taiwan | 2011 | 0456/Taiwan/2011 | 2013 | KC456085 | - | CHG | 3Hv-3 |
| 8 | Taiwan | 2011 | 0552/Taiwan/2011 | 2013 | KC456086 | - | CHG | 3Hv-3 |
| 9 | Taiwan | 2011 | 0736/Taiwan/2011 | 2013 | KC456087 | - | CHG | 3Hv-3 |
| 10 | Taiwan | 2011 | 0889/Taiwan/2011 | 2013 | KC456088 | - | CHG | 3Hv-3 |
| 11 | Taiwan | 2011 | 1012/Taiwan/2011 | 2013 | KC456089 | - | CHG | 3Hv-3 |
| 12 | Taiwan | 2011 | 1112/Taiwan/2011 | 2013 | KC456090 | - | CHG | 3Hv-3 |
| 13 | Taiwan | 2011 | 1133/Taiwan/2011 | 2013 | KC456091 | - | CHG | 3Hv-3 |
| 14 | Taiwan | 2011 | 1225/Taiwan/2011 | 2013 | KC456092 | - | CHG | 3Hv-3 |
| 15 | Taiwan | 2011 | 1301/Taiwan/2011 | 2013 | KC456093 | - | CHG | 3Hv-3 |
| 16 | Taiwan | 2011 | 1332/Taiwan/2011 | 2013 | KC456094 | - | CHG | 3Hv-3 |
| 17 | Taiwan | 2011 | 1444/Taiwan/2011 | 2013 | KC456095 | - | CHG | 3Hv-3 |
| 18 | Taiwan | 2011 | 1697/Taiwan/2011 | 2013 | KC456096 | - | CHG | 3Hv-3 |
| 19 | Taiwan | 2011 | 1735/Taiwan/2011 | 2013 | KC456097 | - | CHG | 3Hv-3 |
| 20 | Taiwan | 2011 | 1764/Taiwan/2011 | 2013 | KC456098 | - | CHG | 3Hv-3 |
| 21 | Taiwan | 2011 | 1854/Taiwan/2011 | 2013 | KC456099 | - | CHG | 3Hv-3 |
| 22 | Taiwan | 2011 | 1962/Taiwan/2011 | 2013 | KC456100 | - | CHG | 3Hv-3 |
| 23 | Taiwan | 2011 | 3434/Taiwan/2011 | 2013 | KC456101 | - | CHG | 3Hv-3 |
| 24 | Taiwan | 2011 | 3440/Taiwan/2011 | 2013 | KC456102 | - | CHG | 3Hv-3 |
| 25 | Taiwan | 2011 | 3759/Taiwan/2011 | 2013 | KC456103 | - | CHG | 3Hv-3 |
| 26 | Taiwan | 1996 | 260-96-TW | 2007 | EF486496 | - | CHG | 3Hv-21 |
| 27 | Taiwan | 1996 | 843-96-TW | 2007 | EF486497 | - | CHG | 3Hv-22 |
| 28 | Taiwan | 1997 | 856-97-TW | 2007 | EF486498 | - | CHG | 3Hv-1 |
| 29 | Taiwan | 1999 | 2127-99-TW | 2007 | EF486499 | - | CHG | 3Hv-1 |
| 30 | Taiwan | 2000 | 16-00-TW | 2007 | EF486500 | - | CHG | 3Hv-23 |
| 31 | Taiwan | 2000 | 4022-00-TW | 2007 | EF486501 | - | CHG | 3Hv-23 |
| 32 | Taiwan | 2002 | 4752-02-TW | 2007 | EF486502 | - | CHG | 3Hv-23 |
| 33 | Taiwan | 2002 | 5276-02-TW | 2007 | EF486503 | - | CHG | 3Hv-23 |
| 34 | Taiwan | 2003 | 1041-03-TW | 2007 | EF486504 | - | CHG | 3Hv-23 |
| 35 | Taiwan | 2005 | 578-05-TW | 2007 | EF486505 | - | CHG | 3Hv-23 |
| 36 | Taiwan | 2002 | 5501-02-TW | 2007 | EF486506 | - | CHG | 3Hv-23 |
| 37 | Taiwan | 1992 | 312-92-TW | 2007 | EF494640 | - | CHG | 3Hv-1 |
| 38 | Taiwan | 1996 | 846-96-TW | 2007 | EF494641 | - | CHG | 3Hv-1 |
| 39 | Taiwan | 1999 | 1964-99-TW | 2007 | EF494642 | - | CHG | 3Hv-3 |
| 40 | Taiwan | 2004 | 1460-04-TW | 2007 | EF494643 | - | CHG | 3Hv-4 |
| 41 | Taiwan | 2000 | 4305-00-TW | 2007 | EF494644 | - | CHG | 3Hv-3 |
| 42 | Taiwan | 2003 | 154-03-TW | 2007 | EF494645 | - | CHG | 3Hv-3 |
| 43 | Taiwan | 2003 | 207-03-TW | 2007 | EF494646 | - | CHG | 3Hv-3 |
| 44 | Taiwan | 2003 | 223-03-TW | 2007 | EF494647 | - | CHG | 3Hv-3 |
| 45 | Taiwan | 2003 | 229-03-TW | 2007 | EF494648 | - | CHG | 3Hv-3 |
| 46 | Taiwan | 2003 | 305-03-TW | 2007 | EF494649 | - | CHG | 3Hv-1 |
| 47 | Taiwan | 2005 | 837-05-TW | 2007 | EF494650 | - | CHG | 3Hv-3 |
| 48 | Taiwan | 2003 | 232-03-TW | 2007 | EF570053 | - | CHG | 3Hv-3 |
| 49 | Taiwan | 1983 | 1179-83-TW | 2007 | EF570054 | - | CHG | 3Hv-2 |
| 1 | **USA** | 1997 | NHRC 1276 | 2004 | AY599836 | [16481660](https://www.ncbi.nlm.nih.gov/pubmed/16481660) | CG | 3Hv-1 |
| 2 | USA | - | human/USA/MEEI_00075/X/3 | 2013 | KF268202 | - | CG | 3Hv-1 |
| 3 | USA | 1953 | GB | 2004 | AY599834 | [16481660](https://www.ncbi.nlm.nih.gov/pubmed/16481660) | CG | GB |
