# Supplementary figures and images for: Worldwide increased prevalence of human adenovirus type 3 (HAdV-3) respiratory infections is well correlated with heterogeneous hypervariable regions (HVRs) of hexon
Source: PLoS One. 2018 Mar 28;13(3):e0194516. doi: 10.1371/journal.pone.0194516 (PMC5874027; doi:10.1371/journal.pone.0194516)

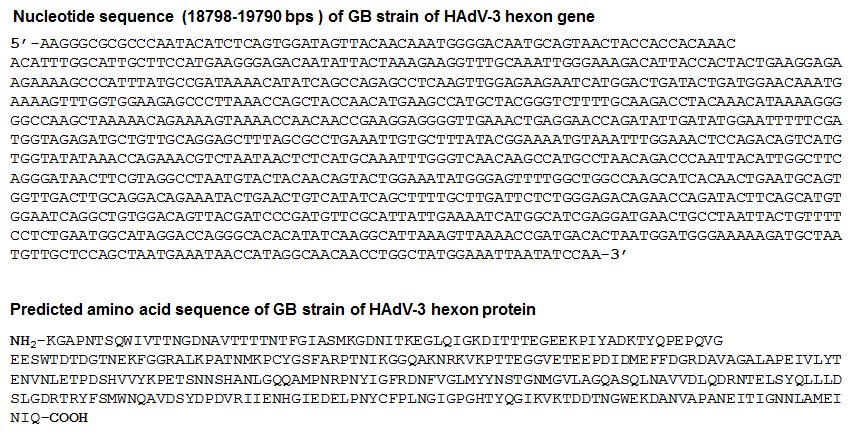

Supplement: S1 Fig — Above: Nucleotide sequence of the GB strain of human adenovirus type 3 (HAdV-3). The hexon gene extends from 18,798 to 19,790 bp (993 bp), a genome sequence that encodes hypervariable regions of the hexon. Below: Predicted amino acid (AA) sequence of the hexon gene sequence mentioned above. The nucleotide sequences were translated to the predicted AA sequences using Genetyx software (Genetyx Corporation, Tokyo). This AA sequence is used as a standard for analysis. (TIF) [file pone.0194516.s002.tif]
